# Patient Experiences and Clinical Outcomes in a Multidisciplinary Perioperative Transitional Pain Service

**DOI:** 10.3390/jpm14010031

**Published:** 2023-12-26

**Authors:** Divya Manoharan, Anping Xie, Yea-Jen Hsu, Hannah K. Flynn, Zodina Beiene, Alexandros Giagtzis, Ronen Shechter, Eileen McDonald, Jill Marsteller, Marie Hanna, Traci J. Speed

**Affiliations:** 1 Department of Psychiatry and Behavioral Sciences, Johns Hopkins University School of Medicine, Baltimore, MD 21287, USA; dmanoha1@jhmi.edu (D.M.);; 2 Department of Anesthesiology and Critical Care Medicine, Johns Hopkins University School of Medicine, Baltimore, MD 21287, USA; axie1@jhmi.edu (A.X.); zodinabeiene@gmail.com (Z.B.); rshecht1@jhmi.edu (R.S.); mhanna9@jhmi.edu (M.H.); 3 Armstrong Institute for Patient Safety and Quality, Johns Hopkins University School of Medicine, Baltimore, MD 21202, USA; jmarste2@jhmi.edu; 4 Department of Health Policy and Management, Johns Hopkins Bloomberg School of Public Health, Baltimore, MD 21205, USA; yhsu9@jhu.edu; 5 Loyola College of Arts & Sciences, Loyola University Maryland, Baltimore, MD 21210, USA; 6 Department of Health, Behavior and Society, Johns Hopkins Bloomberg School of Public Health, Baltimore, MD 21205, USA; emcdona1@jhu.edu

**Keywords:** perioperative pain management, opioids, opioid tapering, multidisciplinary, psychiatry, functional recovery

## Abstract

Siloed pain management across the perioperative period increases the risk of chronic opioid use and impedes postoperative recovery. Transitional perioperative pain services (TPSs) are innovative care models that coordinate multidisciplinary perioperative pain management to mitigate risks of chronic postoperative pain and opioid use. The objective of this study was to examine patients’ experiences with and quality of recovery after participation in a TPS. Qualitative interviews were conducted with 26 patients from The Johns Hopkins Personalized Pain Program (PPP) an average of 33 months after their first PPP visit. A qualitative content analysis of the interview data showed that participants (1) valued pain expectation setting, individualized care, a trusting patient–physician relationship, and shared decision-making; (2) perceived psychiatric treatment of co-occurring depression, anxiety, and maladaptive behaviors as critical to recovery; and (3) successfully sustained opioid tapers and experienced improved functioning after PPP discharge. Areas for improved patient-centered care included increased patient education, specifically about the program, continuity of care with pain specialists while tapering opioids, and addressing the health determinants that impede access to pain care. The positive patient experiences and sustained clinical benefits for high-risk complex surgical patient support further efforts to implement and adapt similar models of perioperative pain care.

## 1. Introduction

Ensuring successful recovery after surgery is critical. Over 100 million surgical procedures are performed annually in the US [1], and approximately 25% of patients undergoing surgery are on preoperative opioids [2,3,4,5,6]. Chronic postoperative pain and long-term opioid use are significant and challenging complications that hinder recovery [7,8]. Preexisting pain [9,10] and use of preoperative opioids [11,12,13] are robust predictors of chronic pain, escalation of opioid use, and impaired recovery after surgery [9,11,12,13,14,15]. Additionally, age, sex, substance abuse, anxiety, and depression increase the risk of poorly managed pain and long-term opioid use following surgery [15,16,17,18]. Furthermore, wide variability in opioid prescribing and care coordination across transitions of the perioperative period increases the risk of opioid chronicity and dose escalation [19,20,21,22,23]. While patients express interest in tapering or discontinuing opioids after surgery, their efforts can be hindered by severe acute postoperative pain, fears of managing chronic pain without opioids, unclear opioid taper plans, and lack of effective communication with their providers [19]. One strategy to improve the delivery of pain care and reduce the risk of high-dose, long-term opioid use for surgical patients includes increased access to a multidisciplinary team of pain and opioid experts who provide personalized multimodal pain management and individualized opioid taper plans that align with patients’ treatment goals and address the underlying conditions that impact chronic pain [8].

Toronto General Hospital pioneered the Transitional Pain Service (TPS) to address gaps in pain management and opioid stewardship across the continuum of perioperative care [24]. Internationally, subsequent programs exist within academic medical centers [24,25,26,27,28] and the United States Veterans Affairs [29] to shepherd high-risk complex surgical patients (e.g., individuals with chronic pain, preoperative opioid use, opioid use disorder (OUD), psychological distress) through three stages of surgical care: preoperative preparation, acute postoperative care, and long-term postoperative recovery. Patients may receive pain psychoeducation, multimodal pain analgesia, opioid tapering plans, and access to non-pharmacological and psychological therapies tailored to their medical conditions at each stage of the surgical period.

The Johns Hopkins Personalized Pain Program (PPP) is a transitional perioperative pain service offering specialized pain management and concurrent psychiatric treatment for patients with preoperative pain and opioid use or those at risk of long-term opioid therapy [26]. Consistent with findings from other institutions [30,31,32,33], the PPP has shown success in facilitating reductions of postoperative opioid consumption with concurrent improvements in clinical outcomes [34,35,36]. Collectively, these data evidence that long-term, multidisciplinary, coordinated pain management adds healthcare value for surgical patients at greatest risk of chronic pain and high-dose long-term opioid use. These outcomes are important; equally important is the pain management experience and quality of recovery from the patient’s perspective. While previous literature has demonstrated buy-in from clinicians for increased access and expansion of transitional perioperative pain services [23,25,28], no study has examined patient experiences with TPSs and patient perceptions of pain and functioning after discharge from transitional perioperative pain services. To address this gap and identify strategies to optimize patient-centered care, this study aimed to examine patient experiences of care delivery in the PPP and perceptions of their pain and quality of life after discharge from the PPP and quantify opioid use from initial PPP treatment to time of interview. 

## 2. Methods

### 2.1. Design

A qualitative study examined patient experiences during and after PPP treatment. Quantitative data from electronic health records (EHRs) were collected for the same patients to assess their baseline characteristics and changes in opioid use over time. The Institutional Review Board of The Johns Hopkins University School of Medicine approved this protocol (IRB00269136) on 26 April 2021, and all participants consented.

### 2.2. Setting

The study was conducted at the PPP, a multidisciplinary transitional perioperative pain service at The Johns Hopkins Hospital in Baltimore, Maryland. A summary of the clinical workflow and outcomes of the PPP has been described previously [34,35]. Briefly, patients eligible for the PPP are surgical candidates on preoperative opioids, opioid-naïve participants at risk of chronic opioid therapy after surgery (e.g., surgery severity, high-dose MME at discharge, psychiatric comorbidities), or patients with a current or past history of OUD. Patients may have surgery at an outside hospital. Surgical candidates who are seen for preoperative evaluation may ultimately not undergo surgery but remain in the PPP. Referrals are made before or after surgery by surgeons, primary care providers (PCPs), other specialists, self-referral, or by the Acute Pain Service consultation service. The care process involves personalized pain education and expectation setting, multimodal analgesia treatment, opioid tapering, and psychiatric co-treatment. Frequency of clinic visits, duration of PPP treatment, and interdisciplinary referrals are tailored to an individual’s pain severity, rate of opioid taper, and co-occurring conditions. For instance, patients at risk of or demonstrating opioid misuse or dependency may have more frequent PPP visits and toxicology screens, be referred to an addiction medicine program for concurrent treatment, or be discharged from the clinic. PPP providers prescribe buprenorphine for patients who have OUD. PPP treatment is complete after patients have tapered opioids to discontinuation or remain on stable low-dose opioids that a PCP or other provider has agreed to prescribe. Participants have the option of continuing care with the PPP psychiatrist beyond the completion of the opioid taper. The PPP provides a warm hand-off to patients’ providers about their PPP treatment and opioid taper plan, especially for out-of-state patients who are often unable to make the commute for long-term PPP care. Patients are verbally told that they are welcome to return to the PPP if they have another surgery or procedure.

### 2.3. Participants

Inclusion criteria for this study included PPP patients who (1) had at least one visit with the PPP, (2) were aged 18 or older, and (3) the date of their first visit with the PPP was at least 6 months prior to the date at which the interview was conducted. The 6-month difference from the first PPP visit to the time of the interview was chosen to collect perspectives from patients in chronic postoperative recovery. Exclusion criteria included PPP patients who (1) were unable to provide oral consent, (2) had active suicidal ideation at study entry, and (3) had a primary psychotic disorder. Eligible participants received up to two email invitations, followed by two phone call requests to participate.

### 2.4. Data Collection

#### 2.4.1. Qualitative Data

Semi-structured interviews. Individual phone interviews were conducted by trained researchers (D.M., H.F., A.G.) between 14 June 2021 and 25 April 2022. To facilitate the interviews, a semi-structured interview guide was developed and pilot-tested (Supplementary S1). The interview guide included questions about (1) general experience with the PPP, (2) the PPP discharge process, including reasons for leaving, and (3) pain experience and pain management since PPP discharge. We intentionally did not ask patients about their current opioid use to encourage candor about their PPP experience and to minimize judgment or stigma about their current opioid use. A larger systematic research study is ongoing to assess long-term opioid use and clinical outcomes following PPP discharge. Some patients who completed care with PPP anesthesiologists and continued to see the PPP psychiatrist were asked about their perceptions and needs associated with eventual discharge. All interviews took 30–60 min and were audio recorded and then transcribed by a researcher for analysis. Personal identifying information was removed from transcripts during transcription.

#### 2.4.2. Quantitative Data

Chart review. At each clinic visit, a PPP physician reviewed the patient’s opioid prescriptions from the EHRs and the Maryland Prescription Drug Monitoring Program (PDMP) and performed a pill count when available. The physician computed and converted the average daily opioid consumption to morphine milligram equivalents (MME) using an opioid conversion application and later recorded in the EHRs [34]. We reviewed the EHRs of all patients participating in the interviews to extract their average daily opioid consumption at the initial and final PPP visits. After completion of the semi-structured interview, we extracted opioid prescription data from Maryland PDMP and converted it into average daily MME. We defined average daily opioid use at the date of the interview as the average daily total MME from opioid prescriptions filled within one month of the interview date.

Patient surveys. Since the inception of the PPP, patients have completed surveys prior to clinic visits regarding their demographic and clinical information (e.g., sex, age, race, education, date and type of surgery, opioid use chronicity, pain chronicity) and self-report questionnaires including the Brief Pain Inventory (BPI) [37], Pain Catastrophizing Scale (PCS) [38], Insomnia Severity Index (ISI) [39], 12-Item Short-Form Health Survey (SF-12 v2) [40], and RAND 36-Item Health Survey (SF-36 v2) [41]. The surveys were administered through Qualtrics from October 2017 to mid-September 2019, at which time the clinic transitioned to REDCap. Patients were emailed a link to the electronic survey one day before their clinic visit. If they could not complete the survey in advance, they completed it at their PPP appointment. Gaps in self-reported clinical information, as well as clinical information about PPP treatment (e.g., PPP visits, treatment duration, physician specialty), were extracted from the EHRs. After each interview, we extracted pain, pain catastrophizing, insomnia, and physical and mental health functioning scores from participants’ first and last PPP visits.

Pain. The BPI [37] was used to assess pain severity and pain interference. Pain severity was calculated as an average of four pain items (i.e., current, general, least, and worst) [42]. Pain interference was calculated as an average of seven items (i.e., relations with others, enjoyment of life, mood, sleep, walking, general activity, and working).

Pain catastrophizing: The PCS [38] was used to assess cognitive and affective responses to pain.

Insomnia Severity: The ISI [39] is a brief, well-validated 7-item survey to assess insomnia.

Physical and mental health: The validated SF-12 v2 [40] was used to measure physical and mental health status from June 2017 through March 2020. The PPP had a license with Optum (Eden Prairie, MN, USA) to administer the SF-12 v2 via the website https://www.amihealthy.com/ (accessed on 23 March 2020). Prior to the COVID-19 pandemic, patients accessed the website on an iPad provided during their in-person clinic visits. In March 2020, the PPP transitioned to telemedicine in response to the pandemic. Since patients could no longer access the website, the PPP transitioned to using the SF-36 v2 [41], which was incorporated into the existing REDCap questionnaire. Eight patients had both SF-12 and SF-36 data.

### 2.5. Data Analysis

#### 2.5.1. Qualitative

A qualitative content analysis of the interview data was conducted [43]. Interview transcripts were reviewed in an iterative process to identify recurring phrases, ideas, and concepts and to create common themes. Interviews and analysis took place in parallel, and interviews were conducted until the study reached saturation, meaning that further interviews did not provide new information [44]. The data analysis process included two main steps: (1) three transcripts were analyzed independently by 2 of 3 team members (D.M., A.X., T.S.) and discussed to achieve consensus, and (2) the remaining transcripts were analyzed by at least 1 team member (D.M., H.F.). Two team members (A.X., T.S.) reviewed and provided feedback on the node structure at each step of the data analysis process. To ensure the quality of data analysis and meet the credibility, dependability, and confirmability criteria, we searched for negative cases to refine the analysis, ensuring that different combinations of team members analyzed each transcript.

Interview analysis showed that participants either described unequivocally overall positive or negative experiences and perspectives of PPP treatment. Thus, team members (D.M., T.S.) categorized participants into two groups: those with positive PPP experience and those with at least one negative PPP experience. Team members achieved consensus among all participants without the need for further discussion. Positive experience was defined as a participant reporting one or more of the following: (1) satisfaction with the program; (2) positive impact on self, family, social, or work relationships associated with PPP participation; (3) positive clinical outcomes associated with PPP participation; (4) praise for PPP providers; and (5) unequivocal willingness to return to PPP if needed. Negative experience was defined as a participant reporting one or more of the following: (1) dissatisfaction with the program; (2) inadequate pain management or negative clinical outcomes associated with PPP participation; (3) self-discharge due to dissatisfaction with the program; and (4) equivocal or lack of willingness to return to PPP if needed.

#### 2.5.2. Quantitative

We described the characteristics of participating patients using frequencies and percentages. We used medians and interquartile ranges to describe these patients’ utilization of PPP, including the number of visits and days between their first and last visits. We then reported and compared pain-related clinical data (e.g., pain severity, pain interference, pain catastrophizing, insomnia severity, quality of life) at the first and last PPP visit and opioid use at the first and last PPP and at the time of interview for all participants and by reported experiences (positive vs. negative) using means and standard deviations. Non-parametric paired Wilcoxon signed-rank tests were used to determine statistical significance between the first and last measures in PPP and at the time of interview (opioid consumption only) within all study patients and in each of the experience groups. Bonferroni analyses were not conducted, given that these ad hoc analyses were exploratory.

## 3. Results

**Figure 1:**
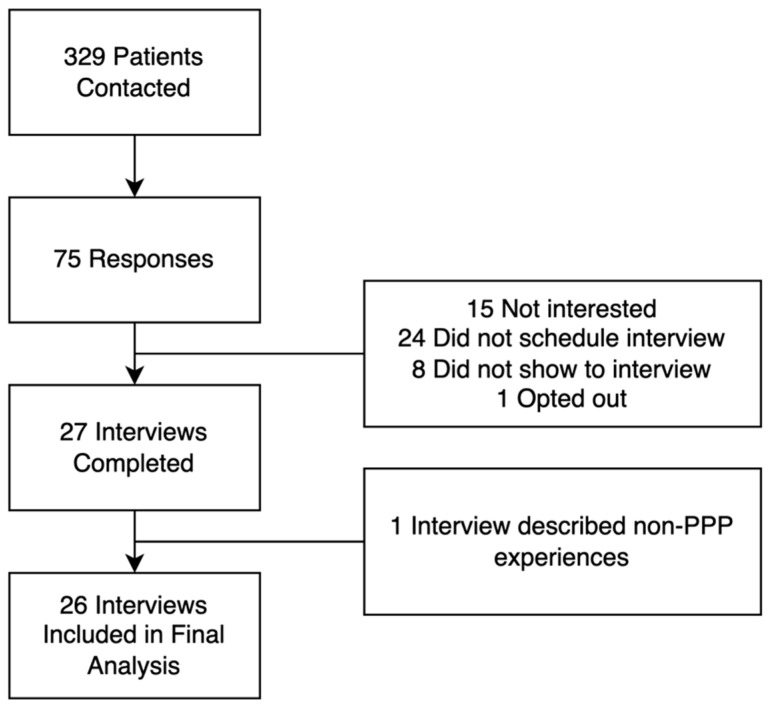
Flow diagram of study participant recruitment, responses, and follow-up.

Figure 1 shows the flow diagram of patient recruitment. We contacted a total of 329 eligible patients, among which 27 completed interviews. One interview, in which the patient described pain care at a different pain clinic, was excluded from the analysis. Table 1 presents the demographic and clinical characteristics of the remaining 26 patients. A total of 20% of respondents were African American, 73% were unemployed or disabled, and 54% were married. In comparison, 37% of the PPP population are African American, 60% are unemployed or disabled, and 36% are married [35]. While approximately 25% of the clinic’s total population had seen the clinic psychiatrist, most respondents in this study (54%) received psychiatric co-treatment. The median number of visits and length of time in PPP care were 8 visits and 196 days, respectively. The median length of time from the first PPP visit to the date of interview was 990 days. Of the 26 interviews, 19 (73%) participants reported positive experiences (i.e., reported that they were satisfied with their care and/or outcomes with the program), and 7 (27%) patients reported negative experiences (i.e., patients were dissatisfied with their care or outcomes and/or self-discharged due to dissatisfaction). There was no statistically significant difference in demographic and clinical characteristics between patients with positive and negative experiences.

## 4. Qualitative Outcomes

Interview data were inductively organized into 7 main themes within 2 phases of care: (1) during treatment (patient understanding and expectations of PPP treatment, patient engagement, psychiatry co-treatment, accessibility and care coordination of pain care); and (2) discharge planning and post-discharge (discharge and care transition, long-term clinical outcomes). While not an a priori outcome of the study, participants shared challenges of living with chronic pain, including experiencing stigma, feeling ignored, limited and delayed access to non-opioid treatment modalities for chronic pain, no guidance on tapering opioids, fearing the process of tapering opioids, and barriers to chronic opioid therapy.

### 4.1. During Treatment

#### 4.1.1. Initiation of PPP Treatment

Two sub-themes emerged related to the initiation of PPP treatment: (1) patient understanding of PPP treatment and (2) patient expectations of PPP treatment (Table 2).

##### Patient Understanding of PPP Treatment

Most participants understood their reasons for referral (Q1). Some were unsure why they were referred to the PPP (Q2). They suggested that the PPP provide a flyer to educate patients on the program’s providers and treatment goals (Q3). Participants also recommended that patients receive an introduction to the PPP and could meet PPP physicians before surgery (Q4).

##### Patient Expectations of PPP Treatment

Most participants entered the PPP expecting to receive pain management and to taper opioids (Q5). Those who were unaware of the PPP’s goal to facilitate opioid tapering suggested that physicians clarify the treatment plan as early as possible (Q6). Some participants had expected more discussion of non-pharmacologic approaches to pain care (Q7).

#### 4.1.2. General Perceptions and Experiences Receiving Care in the PPP

Many participants described the PPP as a unique experience compared with previous pain care (Q8) (Table 3). Participants appreciated access to comprehensive and specialized pain care that extended beyond routine surgical care (Q9). Specifically, participants reported that having a team that could adequately manage postoperative pain helped to reduce the stress of surgery for both the patient and their caregivers (Q10). Most participants highlighted the benefits of multidisciplinary care that addressed both their pain and mental health (Q11).

#### 4.1.3. Patient Engagement

Three sub-themes emerged related to patient engagement: (1) individualized care, (2) patient–physician relationship, and (3) shared decision-making (Table 4).

##### Individualized Care

Participants highlighted the importance of guidance and pain education across the entirety of opioid tapering (Q12). Most participants received a long-term pain management plan that was tailored to their medical conditions (Q13). Several participants emphasized that compared with previous experiences, they did not feel they were stigmatized as drug-seekers (Q14).

##### Patient–Physician Relationship

Participants shared examples of both the cognitive and emotional care components of an effective patient–physician relationship within the PPP. Regarding the cognitive components, participants described the importance of listening to patients (Q15), knowledge and competence in pain medicine (Q16), and clear communication, which helped them understand the opioid taper plan and provided reassurance (Q17). Participants highlighted the value of having family members attend visits because it improved communication with and understanding by their families (Q18).

Participants recommended that they partner with the same PPP physician throughout the opioid taper because they found it difficult to adjust to a new opioid taper plan (Q19) and re-establish the patient–physician relationship (Q20). Participants recommended that if patients needed to transition to a new physician mid-taper that the former physician communicate the plan with the patient and new physician to avoid abrupt changes to the opioid taper pace (Q21).

Regarding the emotional care components of a patient–physician relationship, most patients felt comfortable receiving pain management in the PPP. Participants characterized PPP physicians as supportive, caring, compassionate, and empathetic (Q22). They discussed the importance of feeling respected and trusting their physicians (Q23), which could foster honest discussions with their PPP physician about their opioid use (Q24).

##### Shared Decision-Making

Participants had mixed reports on their capacity to share in decision-making regarding their opioid tapering plan. Some participants described working together with their PPP physician (Q25) and collaborating on an individualized opioid taper plan based on their pain and functioning rather than a specific timeline (Q26).

#### 4.1.4. Psychiatry Co-Treatment in the PPP

Participants described several benefits of receiving psychiatric treatment within the PPP (Table 5). Participants frequently revisited how psychiatric care addressed the whole person (Q27). They highlighted that psychiatry co-treatment was a critical component alongside opioid tapering. Specifically, they described how the treatment of co-occurring psychiatric disorders improved their pain (Q28) and how psychotropics improved their functioning and capacity to return to work (Q29). Participants also described the benefits of having a psychiatrist who could manage substance use relapse (Q30) and help guide them through the rebuilding of interpersonal relationships (Q31). Finally, participants gained newfound confidence in pain self-management non-pharmacological approaches tailored to their preferences, including cognitive behavioral (Q32) and mindfulness techniques (Q33). One participant reported she felt pressured to see the psychiatrist and take psychotropics (Q34), yet overall, participants described how psychiatric care significantly improved the postoperative experience. 

#### 4.1.5. Accessibility and Care Coordination of Pain Treatment

Participants reported challenges in accessing pain management and prescription opioids both within and outside of PPP treatment (Table 6). Participants described the health determinants that limited access to the PPP, including insurance coverage, transportation, and cost of parking (Q35). Those who had received in-person care before the COVID-19 pandemic suggested that the PPP provide telehealth visits (Q36). Participants also described difficulties accessing siloed pain treatments. Specifically, it was challenging for one participant to take additional time off from work to receive injections from a separate interventional pain clinic (Q37).

Participants discussed potential barriers to receipt of prescription opioids. One family member discussed how signing the PPP’s opioid contract would preclude her daughter from receiving opioids from other providers either during an emergency or after PPP discharge (Q38). One participant discussed how individuals may hoard opioid pills after completing opioid tapers because current policies limit access to prescription opioids (Q39). Finally, while one Caucasian participant did not personally experience discrimination, she expressed concern that racism could influence access to prescription opioids for minoritized patients (Q40).

Participants were, overall, satisfied with the coordination of care that the PPP provided. In instances where participants could not go to Johns Hopkins for emergent care, participants appreciated that PPP providers were willing to share pain management recommendations with providers at other medical systems (Q41).

### 4.2. Discharge Planning and Post-Discharge Treatment

#### 4.2.1. Reasons for PPP Discharge and Experiences with Care Transitions

As shown in Table 7, most participants reported that they were discharged from the clinic either when they felt capable of self-managing their pain (Q42) or had follow-up pain management scheduled with another pain clinic or their PCP (Q43). A minority of participants self-discharged from the PPP because they were no longer prescribed opioids or they planned on continuing opioids for active medical conditions (e.g., cancer) (Q44). One participant preemptively self-discharged due to family pressure. She described her partner as being dissatisfied with the care she received and encouraged her to leave the clinic (Q45).

Participants who successfully completed PPP (i.e., physician and patient agreed on discharge) reported satisfaction with the timing and process of discharge. While some participants did not recall a specific discharge process, they denied gaps in pain care (Q46). Participants who could not continue commuting to the PPP discussed how the PPP physicians were willing to manage their pain until they could transition to a local pain clinic (Q47). Some participants continued treatment with the PPP psychiatrist after completing their opioid taper. Among those who self-discharged from the PPP, they found it difficult to obtain opioids from other providers (Q48).

Many patients were willing to return to PPP for subsequent surgeries. Participants felt reassured by the knowledge that PPP was a resource they could return to for perioperative pain management if necessary (Q49).

#### 4.2.2. Long-term Outcomes

##### Clinical Outcomes Associated with PPP Treatment

Overall, participants described marked improvements in five domains: (1) reduced pain, (2) improved physical health, (3) improved mental health, (4) the ability to return to work, and (5) improved relationships (Table 8). Most participants associated PPP treatment with both improved quality of life and pain reduction (Q50). Some participants with a history of chronic pain were surprised to experience days without pain (Q51). Participants described how treatment of depression and anxiety improved their pain (Q52). Similarly, insomnia treatment improved their quality of life (Q53). Many participants were still practicing pain self-management techniques that they learned from the PPP, including distraction and non-pharmacological treatments (Q54). One participant who learned about the bidirectional relationship between pain and mood is using mind and body techniques as she continues to taper off opioids (Q55).

Participants also discussed notable ways that discontinuing opioids has contributed to their improved quality of life (Q56), including lifting brain fog and increasing cognitive functioning (Q57). This same participant described his wife’s positive reaction to his opioid discontinuation (Q58). Another participant described how opioid discontinuation had improved her relationship with her family (Q59). One participant described that since discontinuing opioids, she was no longer distracted at work by an urge to use them (Q60). Conversely, some participants found that discontinuing opioids worsened their quality of life and that resuming low-dose opioids increased their capacity to return to work and function (Q61).

##### Treatment Satisfaction

**Figure 2:**
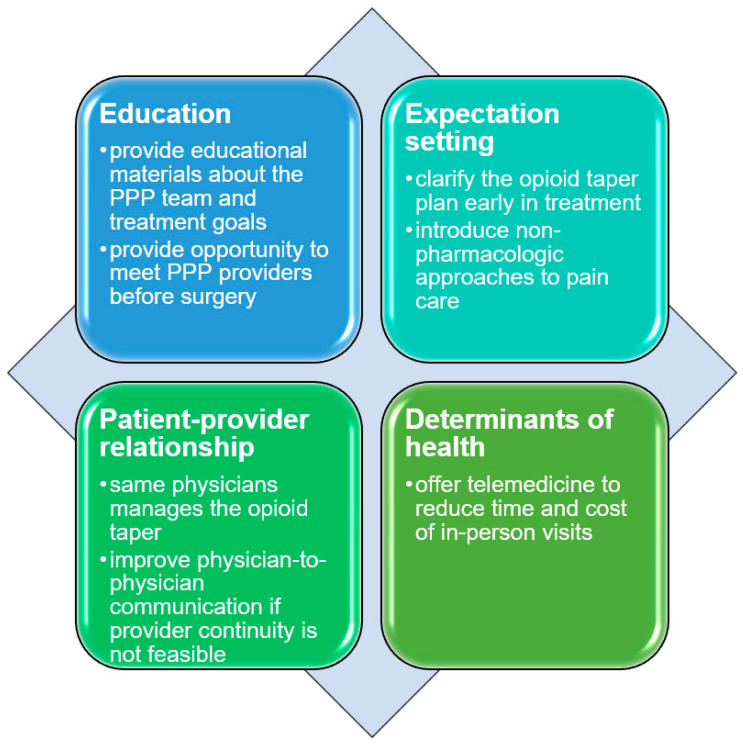
Participant recommendations to improve patient-centered perioperative pain care.

Nearly three-quarters of participants reported overall satisfaction with PPP treatment. Participants described that the PPP met their expectations (Q62) and expressed gratitude for the program’s effect on their quality of life (Q63). Although participants were largely satisfied with their care in the PPP, they offered the following recommendations to enhance patient-centered care, as summarized in Figure 2.

### 4.3. Quantitative Outcomes

#### 4.3.1. Opioid Use

Among all participants (Table 9), opioid use was significantly reduced between the first and last PPP visit (*p* = 0.004) and between the first PPP visit and the time of interview (*p* = 0.03). Opioid use did not significantly change between the last PPP visit and the time of interview (*p* = 0.96). A review of PPP clinical data and PDMP data showed that 11 participants had discontinued opioid use by PPP discharge, and 13 (50%) had discontinued or maintained discontinuation of opioids at the time of interview.

Participants with a positive PPP experience significantly reduced opioid use between the first and last PPP visit (*p* = 0.02) and between the first PPP visit and time of interview (*p* = 0.05) and did not have significant changes in opioid use between the last PPP visit and time of interview (*p* = 0.66). Those who reported a negative PPP experience had a nonsignificant reduction in opioid use from the first to last PPP visit (*p* = 0.09) and no significant change in opioid use between the first PPP visit and time of interview (*p* = 0.29) and between the last PPP visit and time of interview (*p* = 0.40).

#### 4.3.2. Patient-Reported Outcomes

As shown in Table 10, among all participants, pain interference significantly improved from the first to last PPP visit (*p* < 0.001). No other outcomes changed significantly. 

In an ad-hoc comparison of participants who described positive versus negative experiences, among those with a positive experience, mental health functioning at the first PPP visit was significantly higher (*p* = 0.04). Participants who reported a positive experience had less pain severity (*p* = 0.02), less pain interference (*p* = 0.02), and less pain catastrophizing (*p* = 0.02) at the last PPP visit, and higher mental health functioning (*p* = 0.02) based on the last SF-12 survey, compared with those who reported a negative experience.

## 5. Discussion

To our knowledge, this is the first study examining patients’ experiences with treatment in a transitional perioperative pain service that provides concomitant pain and psychiatric care. This study demonstrated participants’ perceived value of long-term specialized, coordinated, and individualized care during the perioperative period that facilitated opioid tapering while addressing both pain and the psychiatric conditions that impact surgical recovery. Specifically, participants described that personalized, multidisciplinary perioperative pain care facilitated sustainable opioid discontinuation or reduction, reduced pain, improved mood, sleep, and cognition, increased their capacity to return to work, enhanced interpersonal relationships, and helped manage maladaptive behaviors after surgery.

The establishment of an effective patient–physician relationship, which includes both emotional (e.g., trust, empathy, respect, acceptance) and cognitive (e.g., expectation management, patient education, communication) care components, is essential in pain management [45]. The shifting responsibility of opioid prescribing across the transitions of perioperative care between surgeons and primary care providers [19,46,47,48] exacerbates the difficulties of effective relationship building in pain management [49]. The process of tapering patients’ long-term opioids can also fracture the patient–provider relationship [50,51]. Our qualitative analysis evidenced that pain specialists established effective patient–physician relationships while facilitating opioid tapering. These findings complement evidence that individualized care, validation of pain experiences, and shared decision-making during the opioid tapering process promote success [52,53,54] and suggest that access to the transitional perioperative pain care model may address gaps in routine care that hinder opioid tapering. Participants also described how engaging families in PPP treatment provided an opportunity to educate caregivers about multimodal analgesia and address their concerns; however, family members also dissuaded participants from continuing treatment, specifically tapering opioids. This highlights the complexity of family dynamics on the pain experience [55,56] and may partly explain our previous findings that family engagement was not significantly associated with opioid tapering after surgery [35].

We have previously demonstrated that transitional perioperative pain services have the potential to facilitate opioid reductions during treatment [34,35,36,57]. To our knowledge, this is the first study to report sustained reductions in opioid use, in terms of the number of participants who discontinued opioids and mean opioid doses, nearly three years after initiating treatment in a transitional perioperative pain service. Although this is a small qualitative study, this finding is still important; it demonstrates a new clinical care pathway that has the potential to address the opioid crisis more broadly by facilitating sustainable opioid reductions after surgery. While the limited effectiveness of chronic opioid therapy and the benefits of tapering opioids are well-established, patient fears about and negative experiences with opioid tapering persist [58,59,60,61]. This study contributes to the growing literature on the long-standing benefits of opioid tapering, such as improved pain, physical and mental health, capacity to work, and interpersonal relationships [62,63,64,65], and demonstrates that patients have experienced potentially enduring benefits of initiating opioid tapering in the perioperative period.

A finding unique to this study is the perceived value of concurrent psychiatric treatment, with participants repeatedly highlighting this medical specialty as critical to their long-term recovery after surgery. The PPP is the first transitional perioperative pain service to include psychiatrists rather than psychologists [24,25]. While it is well known that psychiatric disorders (e.g., depression, anxiety, substance use disorders, posttraumatic stress disorder) are common in chronic pain, often they are not recognized or, when recognized, undertreated [66]. Psychiatric comorbidities are associated with increased opioid use, surgical complications, and decreased likelihood of post-surgical opioid cessation [67,68,69]. Tapering opioids has also been associated with the onset or exacerbation of anxiety, depression, and aberrant drug use [50,70,71,72]. We found that participants comprehended the bi-directionality of their pain and mental health and attributed the initiation or modification of psychotropics and access to psychotherapy as crucial contributions to improved pain, physical function, mood, sleep, and cognition. Moreover, participants welcomed individualized therapeutic approaches that facilitated self-efficacy, re-established relationships with family, and helped modify maladaptive pain behaviors. Notably, participants did not spontaneously identify the stigmatization of psychiatric care, a well-known challenge in accessing psychiatric treatment, as a barrier to care. Taken together, patients accepted and benefited from the integration of psychiatry into perioperative pain care.

While most participants perceived PPP as a positive and helpful treatment, several key growth areas emerged from this study’s findings. Since most patients commence PPP treatment postoperatively, participants requested to meet the PPP treatment team and learn more about the program’s patient population and treatment goals before surgery. Although the evidence of educational interventions on pain- and opioid-related outcomes are mixed [73,74], these materials are important to patients and should be part of patient-centered perioperative care. An ongoing research study (clinicaltrials.gov, NCT05252767) will shed light on whether educational materials can improve knowledge and understanding about the PPP and subsequently facilitate improvements in patient engagement during the opioid tapering process.

Participants also recommended that physicians clarify the opioid taper plan as early as possible and introduce more non-pharmacologic approaches to pain care. Readiness to taper opioids is associated with a more positive opioid tapering experience [75,76]. Consistent with previous findings, we found that participants who expected multimodal analgesia and postoperative opioid tapering, or those who welcomed those expectations early in treatment, had better treatment experiences. Experiences with opioid tapering are highly variable. Further quality improvement approaches are warranted to optimize patient-centered individualized approaches to perioperative pain management, including dedicating more time and resources to pain expectation setting, optimizing pharmacological and non-pharmacological multimodal analgesia, facilitating a slower opioid taper, providing early access to mental health care, or expanding access to other behavioral interventions. 

Tapering opioids is a vulnerable experience in part due to stigmatization associated with chronic opioid use, discomfort from opioid withdrawal, mood and pain fluctuations, and uncertainty of the quality of life without opioids [58,59,60,61]. Importantly, we found that participants preferred to undergo the process of opioid tapering with the same physician. Participants described how difficulty adjusting to a new physician’s management plan and opioid tapering expectations negatively impacted their treatment experience. This is a consequential finding because it sheds light on the discrepancies between patient and physician priorities: patients prioritized the continuity of a well-established trusted patient–physician relationship, while PPP physicians prioritized facilitating opioid tapering regardless of provider. This finding also supports the need for the widespread adoption of specialty perioperative pain services, which, as mentioned previously, can lessen the variation and uncertainty of opioid prescribing responsibility between surgeons and other providers [77], thus aligning with patients’ preferences and increasing the likelihood of successful opioid tapering. Taken together, efforts to improve patient-centered care should include strategies to maintain stable patient–physician relationships and improve physician-to-physician communication if provider continuity is not feasible.

Fourth, participants reported how determinants of health, such as cost of parking, availability of transportation, and commute time, negatively affected their experience. Participants recommended telemedicine to increase PPP accessibility, especially for patients who had difficulty ambulating after surgery or lived farther away. These interviews were conducted with participants who were initially seen prior to the COVID-19 pandemic. The PPP transitioned to telemedicine services in March 2020 [78]; ongoing studies are evaluating how this transition has impacted clinical outcomes [57] and patients’ experiences in the PPP.

Our findings must be considered in the context of study limitations. Since interviews were conducted between 6 months and 3 years after treatment, participants may have experienced recall bias. We had a low response rate, which left our data vulnerable to only capturing a subset of patient experiences within the clinic. Our sample overrepresents patients who received psychiatric co-treatment, and although some participants had OUD, they were underrepresented in this study. We completed study enrollment once qualitative analyses reached saturation. Thus, the sample size was too small to conduct Bonferroni analyses to determine baseline or clinic differences between participants with positive and negative treatment experiences (not an a priori study aim). Larger studies are needed to determine if baseline measures such as pain catastrophizing (a robust predictor of pain outcomes), treatment duration, or psychiatric co-treatment, which were not significantly different in this small cohort, are associated with positive versus negative treatment experiences. The small sample size and missing data also precluded analysis of patient engagement and satisfaction outcomes during PPP treatment. Finally, it is well-known that lower participation rates among minority populations is an ongoing problem. While one-fifth of study participants were African American, our findings likely inadequately represent the experience of minority participants undergoing opioid tapers, especially given well-established health inequities in pain management [79,80]. Balancing these limitations, these qualitative data still provide clear directions for quality improvement efforts specific to postoperative pain and opioid management.

In summary, patient stakeholders perceived access to pain experts and psychiatrists as fundamental to their post-surgical recovery and long-term well-being. Given that transitional perioperative pain services are valued by patients and may successfully and sustainably improve surgical outcomes for high-risk complex patients, further considerations are needed to facilitate the implementation and adaptation of similar care models that align with patients’ goals and values.

## Figures and Tables

**Table 1:** Patient baseline demographics and clinical characteristics at initial PPP visit and PPP treatment characteristics.

	All (n = 26)		Negative Experience (n = 7)		Positive Experience (n = 19)		*p* Value
Age, median (IQR)	43.5	(37–56)	56	(46–60)	41	(35–50)	0.06
Sex, n (%)							
Female	20	76.9	5	71.4	15	78.9	1.00
Male	6	23.1	2	28.6	4	21.1	
Race							
White	21	80.8	5	71.4	16	84.2	0.59
Black	5	19.2	2	28.6	3	15.8	
Marital status, n (%)							
Single	10	38.5	4	57.1	6	31.6	0.41
Married	14	53.9	3	42.9	11	57.9	
Separated/Divorced/Widowed	2	7.7	0	0	2	10.5	
Education, n (%)							
Below college	16	61.5	5	71.4	11	57.9	0.67
College degree or high	10	38.5	2	28.6	8	42.1	
Employment, n (%)							
Employed	7	26.9	3	42.9	4	21.1	0.34
Unemployed/Disabled/Retired	19	73.1	4	57.1	15	79.0	
Insurance, n (%)							
Private	18	69.2	5	71.4	13	68.4	1.00
Public	8	30.8	2	28.6	6	31.6	
Diagnosis of mood disorder, n (%)							
No	19	73.1	6	85.7	13	68.4	0.63
Yes	7	26.9	1	14.3	6	31.6	
Diagnosis of anxiety, n (%)							
No	22	84.6	7	100.0	15	79.0	0.55
Yes	4	15.4	0	0	4	21.1	
Surgery, n (%)							
Cardiac/Thoracic	2	8.7	1	14.3	1	6.3	0.84
GI/Abdominal	5	21.7	1	14.3	4	25.0	
Neuro/ENT	1	4.4	0	0	1	6.3	
Ortho/Trauma	11	47.8	3	42.9	8	50.0	
Plastic/Vascular	4	17.4	2	28.6	2	12.5	
No. of PPP visits, median (IQR)	7.5	(2–21)	4	(2–10)	8	(2–30)	0.30
No. of anesthesia visits, median (IQR)	2	(2–7)	4	(2–8)	2	(1–7)	0.30
No. of psychiatrist visits, median (IQR)	1	(0–8)	0	(0–2)	3	(0–20)	0.18
Duration of PPP visits, median (IQR)	196	(28–833)	103	(28–294)	252	(21–1050)	0.34
Duration of anesthesia visits **, median (IQR)	42	(14–217)	103	(28–245)	32	(0–210)	0.36
Duration of psychiatrist visits **, median (IQR)	472.5	(161–997.5)	123	(70–175)	819	(224–1029)	0.13
From Wilcoxon rank sum tests or Fisher’s exact tests							

** Only those who had anesthesia or psychiatrist visits were included. There were 25 (96%) patients who had at least one anesthesia visit and 14 (54%) patients who had at least one psychiatrist visit.

**Table 2:** Participant understanding and expectations of PPP treatment.

Nodes	Quote	Participant (Age, Race, Sex)
Patient understanding of PPP treatment		
Understood reason for referral	Q1: “When I first heard about it, it struck me as a highly compassionate and necessary program. Because there are plenty of former opioid users who need surgery and who need to go on opioids to manage their pain. And I think it’s just a brilliant concept.”	P12 (57, C, F)
Unclear of reason for referral	Q2: “I guess sometimes I was a little confused about the, I don’t know, the goals of the program or the types of patients they serviced… do I even deserve to be here and be taking up these people’s time.”	P10 (37, C, F)
Improve patient education about the PPP	Q3: “… a flyer explaining what this perioperative pain clinic is and the types of specialists you’ll encounter, and the goal of the program, too.”	P10 (37, C, F)
Meet PPP team before surgery	Q4: “Once it’s determined that, look, you’re going to have this surgery, maybe that’s an option for them. We’d like to ask you a few questions and see if you wouldn’t want to get involved, or meet with that pain team.”	P15 (52, C, M)
Patient expectations of PPP treatment		
Multimodal pain care	Q5: “I was already on high doses of narcotics and…desiring to come off of them… [I expected] weaning [and] adding on multimodal methods of pain management at the same time.”	P14 (43, C, F)
Set opioid taper expectations early	Q6: “Like, OK, we’re going to prescribe you narcotics for 30 days, then we’re going to move into other approaches that might be helpful to you. Letting people know what to expect so that there aren’t any surprises.”	P11 (60, C, F)
More non-pharmacologic approaches	Q7: “I feel like it might be nice [to have a] discussion of spectrum of services available. [The PPP] focuses mainly on medications… Here are some non-medication related therapies, like massage or acupuncture or things like that.”	P05 (54, C, F)

Q: Quote; P: Participant; C: Caucasian; M: Male; F: Female.

**Table 3:** General perceptions and experiences receiving care in the PPP.

Nodes	Quote	Participant (Age, Race, Sex)
Unique pain experience	Q8: “When I got into the PPP, the care that I received was so different from anything else that I had ever experienced.”	P07 (72, C, M)
Preferred having pain specialist	Q9: “It was really beneficial to feel like I had a physician keeping close watch over me and my recovery who was a bit more of a specialist than my [primary care provider] would have been.”	P10 (37, C, F)
Access to pain experts reduced stress	Q10: “It was going to put my mind at ease. Reducing the stress going into surgery, I think, not only my quality, but my wife’s quality, who’s my caretaker. Knowing, her peace of mind, and knowing that we’ve got a team here that’s solely going to watch my pain and manage it appropriately. So, I mean, I think…that peace of mind is immeasurable, really.”	P15 (52, C, M)
Benefits of pain and mental health co-treatment	Q11: “My experience had been honestly out of this world for my mental health, you know, to my pain and managing my mental health, you know, and managing my pain through my mental health.”	P24 (45, AA, F)

Q: Quote; P: Participant; AA: African American; C: Caucasian; M: Male; F: Female.

**Table 4:** Patient engagement during PPP treatment.

Nodes	Quote	Participant (Age, Race, Sex)
Individualized Care		
Pain education continued throughout opioid taper	Q12: “[There] was always somebody kind of guiding you… It was helpful to have somebody there with you… reassuring you that it’s a gentle and gradual process, and that you will feel uncomfortable during the process of weaning off of your medications.”	P14 (43, C, F)
Pain care tailored to individual’s medical history	Q13: “It put my mind at ease that somebody was monitoring what was going on, somebody knew exactly what has worked, hasn’t worked, and came up with a game plan. [It helped with] reducing the stress going into surgery.”	P15 (52, C, M)
Reduced stigma about opioid use	Q14: “I was no longer treated as a drug seeker. I mean, nothing infuriated me more than that… when I was dealing with physicians and surgeons.”	P07 (72, C, M)
Patient–physician relationship		
Listened to patients	Q15: “I felt like [the physician] really took the time to just hear my whole [story], it’s a complicated thing.”	P13 (30, C, F)
Knowledgeable about pain	Q16: “I think that for the types of therapy that were offered, that people seemed knowledgeable.”	P05 (54, C, F)
Clear communication	Q17: “The pain management team [helped us] better understand what was going on, and to come up better game plan and something that was consistent, and I thought that that was a big help.”	P15 (52, C, M)
Family engagement	Q18: “You know, my husband came with me quite a few times for visits, and it was nice that he could be there and ask questions himself… And I appreciated because it was easier to explain and to be heard from my family since they knew this was happening. I would definitely say it was good…to bring in a family member with me, that they would be able to get the same information and understanding.”	P16 (44, C, F)
Challenges adjusting to new physician’s opioid taper plan	Q19: “After a couple of visits with the original doctor, we agreed according to my history and so on and so forth, that it was gonna take time and we were gonna do a mutually-agreed upon step-down… and then maybe the third visit I see someone different… and [they] said [they] thought I could handle larger step-downs… and that becomes a totally different situation, which wasn’t what I was led to believe I was getting into.”	P03 (60, C, M)
Challenges establishing patient–physician relationship with new physician	Q20: “I wish I had kind of stayed with [my first PPP physician]. Because then I felt like I had to re-explain everything from the beginning with [my new physician]. And I didn’t necessarily feel that same comfort level… I felt like I was kind of like jumping past go, you know, without really, really looking at the whole picture.”	P13 (30, C, F)
Patients prefer continuity of care with same physician	Q21: “I think it’s always nice if there’s continuity in care with physicians… I had a very nice doctor, and [they] refilled my prescription for the pain meds I was using. Then I was set for a second appointment to come back, and I saw a different doctor… and [they] did not extend my prescription… I think that, if that was the intent, that the first doctor should have told me, okay, we’re going to give you one prescription and move forward from there.”	P11 (60, C, F)
Qualities of physician	Q22: “You guys by far have the best doctors there. They are empathetic, the compassion… it really showed, you know, that they care.”	P24 (45, AA, F)
Trust	Q23: “I owe the pain center… an enormous debt for this. I look forward to our appointments. I find [the physician] just delightful as a doctor and a person. And I really trust [them].”	P12 (57, C, F)
Honesty	Q24: “I was always honest with them… if you’re not honest with them I mean they really can’t help you so you got to be honest. I was honest with them and they made sure to not give me too much or too little.”	P18 (30, C, M)
Shared decision-making		
Patient and physician partnered together	Q25: “[The PPP physician] worked with me in helping me manage the postoperative pain and weaning me down from what they had me on—without being judgmental.”	P08 (49, C, F)
Individualized opioid taper plan	Q26: “There was a lot of compassion, and also I think the general tone of working with the patient, and not pushing too hard, trying to do within a reasonable amount of time, and working with the patients, I guess, what’s the word, tolerance, in terms of pain levels, what a patient can tolerate day to day and still function. And talking about that.”	P03 (60, C, M)

Q: Quote; P: Participant; AA: African American; C: Caucasian; M: Male; F: Female.

**Table 5:** Experiences receiving psychiatry co-treatment in the PPP.

Nodes	Quote	Participant (Age, Race, Sex)
Whole person approach	Q27: “A lot of doctors, they gloss over, if you say well this is happening, and they just don’t understand how much it is affecting your life. I think that [my psychiatrist has] given me a lot of encouragement and support, and I truly appreciate that.”	P16 (44, C, F)
Treating anxiety improves pain	Q28: “[Psychiatrist] put me on a mood stabilizer, which is not something I had ever tried. It’s completely affected my life in the fact that it stopped that really bad anxiety feeling that was causing me incredible pain.”	P20 (41, C, F)
Psychotropics improved capacity to work	Q29: “I couldn’t have made it through surgery without it. [The psychiatrist] got me back on antidepressants and anti-anxiety medications. And that has made a huge difference for me. After being unemployed for a good while, like 9 months, I got a great job that I love.”	P12 (57, C, F)
Management of substance abuse	Q30: “I think I had one relapse, but it was very short, and it was a one-time thing, and it was impulsive, and I can be a little impulsive… [the psychiatrist] dealt with that pretty well.”	P12 (57, C, F)
Interpersonal relationship building	Q31: “I’ve really tried hard to maintain relationships and repair them. And I’m a very, very lucky woman. And again, I owe the pain center and [my psychiatrist] an enormous debt for this.”	P12 (57, C, F)
Cognitive behavioral therapies	Q32: “Ways of being, ways of behaving, ways of thinking. So, it wasn’t as simple as they got my narcotic level down…it was really gaining the emotional strength I’d completely forgotten I had.”	P07 (72, C, M)
Mindfulness therapies	Q33: “Stretches daily, in the morning and throughout the day. Mind over matter that we talked about. When the pain really gets bad, have a meditation moment. Being more aware of my environment, sounds, smells, trying to tune out the pain.”	P00 (45, AA, F)
No interest in mental health treatment	Q34: “I kept getting pushed onto mental health and everyone saying that I needed antidepressants and anti-anxiety and no one would listen to me when I’m like that isn’t the problem. I don’t have those issues.”	P25 (49, C, F)

Q: Quote; P: Participant; AA: African American; C: Caucasian; M: Male; F: Female.

**Table 6:** Accessibility and care coordination of pain treatment and opioids.

Nodes	Quote	Participant (Age, Race, Sex)
Accessibility of pain treatment and opioids		
Health determinants limited pain care access	Q35: “The biggest downside that I had was just getting access to the clinic itself in the building. I had to pay about 15 dollars for parking every time I came. I added up to 60 dollars a month for the parking there.”	P05 (54, C, F)
Recommend telehealth to increase access	Q36: “Now in this day and age, [it would help if there were] options for telehealth visits.”	P01 (36, C, F)
Difficulty accessing multiple pain clinics	Q37: “For me, the only issue I had was scheduling conflicts. If I wanted to get an injection, it was hard to get an injection with my work schedule.”	P00 (45, AA, F)
Fears that PPP opioid contract would limit access to opioids	Q38: “They had [the patient] sign paperwork saying she wouldn’t see another doctor… And when we tried to reach back out it was challenging to understand if [the patient] could go back to her primary care physician or not. So I think that legality around prescribing narcotics caused a little bit of a challenge there because of the impact to her other medical problems.”	P13 (30, C, F)
Patients may not dispose of opioids	Q39: “Do they keep their pain medication after their treatment? We agreed that storing up of medication is something that some people felt an impulse to do. I think that some of the safeguards also cause the hoarding of medication.”	P05 (54, C, F)
Biases may influence opioid prescribing	Q40: “I’m a white woman and its easier for me to get pain medication, white privilege and systemic racism. Somebody who’s a young man of color may have a hard time getting pain medication if he needed it.”	P05 (54, C, F)
Care coordination of pain treatment and opioids		
Physicians coordinated care with other health systems	Q41: “Before I went back for my follow up, I ended up going into an emergency. I went to another hospital that was closer at the time because of the amount of pain I was in. And when I told [the PPP] what had happened, they were like, Oh my God. And they called the hospital that was working with me, and they got the instruction. So, to me that was positive.”	P22 (40, AA, F)

Q: Quote; P: Participant; AA: African American; C: Caucasian; F: Female.

**Table 7:** Reasons for PPP discharge and experiences with care transition.

Nodes	Quote	Participant (Age, Race, Sex)
Reasons for PPP discharge		
Able to self-manage pain	Q42: “I just managed it on my own. I definitely didn’t transition to anyone else for that pain. As soon as that surgical pain felt like it was manageable, I stopped going to the clinic and I didn’t see anyone else for that particular pain.”	P10 (37, C, F)
Transitioned to local pain clinic	Q43: “Just by trying to help me find a place closer to home that would try and help me. And [the physician] stuck with me until we did, [they] didn’t just drop me.”	P08 (49, C, F)
Planned to continue opioids for cancer-related pain	Q44: “I also knew that the radiation oncologist was probably going to put me back on pain medication, even if I got off of [opioids] in what was a very short time frame of… so that was a big ask.”	P03 (60, C, M)
Family member encouraged patient to discharge	Q45: “[I thought there would be] different medications that [the physician] was going to put me on that might be able to relieve my pain better… my boyfriend ended up going down with me, and he knew my history with my injuries and stuff like that… he didn’t think it was great, and he was the one who told me not to go back.”	P06 (35, C, F)
Experiences with care transitions		
No gaps in pain management	Q46: “I honestly can’t remember how I was quite discharged from the clinic. If it was like “call us if you need us” or what, I can’t remember how that relationship formally ended. But I don’t remember leaving there feeling high and dry or anything.”	P10 (37, C, F)
Providers coordinated a warm hand-off with a local pain clinic	Q47: “I’m in a regular pain management program now, eventually trying to wean me off the last of it but I’ve had a few problems with the back surgery since then. But [the PPP] helped me find a long-term pain management doctor down here since I was no longer able to participate in the program.”	P08 (49, C, F)
Difficulty obtaining opioids after PPP discharge	Q48: “I ended up having to then get an appointment with my doctor up here to get pain meds and then they don’t want to prescribe them, and they want [the PPP] to prescribe them and [the PPP] won’t prescribe them and it’s an awful stressor that I just don’t think keeps the patient at heart.”	P11 (60, C, F)
Reassurance that PPP is an accessible resource	Q49: “That’s something else I got out of the program, was the reassurance that if [stuff] happened, and we all know that it does from time to time, that I had a resource that I could reach for, that I didn’t have to go through the whole thing of who I am, what I am, where I’m at. That kind of understanding… was really wonderful.”	P07 (72, C, M)

Q: Quote; P: Participant; C: Caucasian; M: Male; F: Female.

**Table 8:** Clinical outcomes associated with PPP treatment and treatment satisfaction.

Nodes	Quote	Participant (Age, Race, Sex)
Clinical outcomes associated with PPP treatment		
Improved quality of life	Q50: “[The PPP] has made my life significantly better. I am not pain-free with surgery-related pain, but it’s significantly improved, and also my sort of overall quality of life has seen a drastic improvement.”	P02 (38, C, F)
Pain reduction	Q51: “I never knew that I would be able to have a life with days without pain… I swear you wouldn’t have told me in a million years that I would be in this place with my pain.”	P24 (45, AA, F)
Treating anxiety and depression improved pain	Q52: “You know, managing my depression and anxiety has also had a positive side effect on my pain.”	P12 (57, C, F)
Treating insomnia improved quality of life	Q53: “I was able to sleep, which is important for quality of life.”	P05 (54, C, F)
Learned to practice pain self-management	Q54: “I am a really big believer in distractions. So, I have hobbies… but, if those things aren’t working, then I will either go to heat, or ice, or massage, or something to try to, you know, relieve some of it. I have physical therapists that I go to on a semi-regular basis.”	P16 (44, C, F)
Learned about bidirectional relationship between mood and pain	Q55: “One thing I learned about my pain, too, when you sit in it and you dwell in it to me… It gets worse. So, I do try to get up. I try to move around. You know, a lot of self-love, a lot of self-everything. So, I do a lot of that every day, [so] I can get off these meds.”	P24 (45, AA, F)
Sustained opioid taper improved quality of life	Q56: “They helped me wean off and see exactly where my threshold was, you know. Being bogged down with all that medicine, you can’t think, and you can’t function the way you need to… I had a cocktail of medicines that I’d been on for a really long time, and [the physician] was able to switch them up, and I have seen a lot of difference between when I first started there and now.”	P16 (44, C, F)
Opioid discontinuation improved cognition	Q57: “The pain program has been transformational. This whole piece of regaining some of that lost functioning was borderline miraculous. I could feel myself coming out of a fog that, although I kind of knew I was in, I had no clue as to how deeply I was embedded in that fog, confusion, lack of concentration.”	P07 (72, C, M)
Family observed improved functioning after opioid discontinuation	Q58: “I said something to the effect of… ’Do you see some kind of difference?’ And she said, ‘Absolutely.’ And I said, ‘Well, I assume emotionally? I’m conveying an improvement?’ And she said, ‘Definitely. As a matter of fact, the way you are now is much more like the man I married.’”	P07 (72, C, M)
Opioid discontinuation improved interpersonal relationships	Q59: “It changed how my family felt about me, they were so excited to have me be free. I’ll never be free from pain but free from opioids.”	P17 (74, C, F)
Opioid discontinuation improved work capacity	Q60: “In the infancy of having the pain, and being on the narcotics, it was very easy to have the knee-jerk reaction of I have to take medication, I have to go home. But I can’t go home for work, I have to hold down a job… my quality of life is much improved because I’m off of the narcotics.”	P14 (43, C, F)
Improved functioning with low-dose opioids	Q61: “And the fact that I wasn’t taking any pain medication at all made it difficult to sleep, aid my family life, it was difficult, and work was very difficult. And I needed to at least find a functional level. I’m not happy about it; it slows me down, it affects everything that I do; the level of medication I’m on. But, I am functioning better than I was without medication at all; I still have pain every day.”	P03 (60, C, M)
Treatment satisfaction		
Met expectations	Q62: “It generally improved my quality of life because it achieved what it was supposed to do, which was get me off of the pain medication.”	P14 (43, C, F)
Gratitude for program	Q63: “I have had a very good experience from the pain management part to the therapy I’m getting with [the psychiatrist]…I have had this chronic illness for 23 years now… and this is the only plan that has worked for me…So, I am very grateful and I’m very appreciative and I always tell you know…if it wasn’t for [them], you know, I really don’t know where I would be.”	P24 (45, AA, F)

Q: Quote; P: Participant; AA: African American; C: Caucasian; M: Male; F: Female.

**Table 9:** Opioid use in MME at first PPP visit, last PPP visit, and time of interview, with a two-group comparison between participants with positive and negative PPP experiences and pre-post comparisons for all participants, those with negative experiences, and those with positive experiences.

	All Study Participants		Negative Experience		Positive Experience		*p*-Values			
								Pre-Post Comparison		
	N	Mean ± SD	N	Mean ± SD	N	Mean ± SD	Two-Group	All	Neg	Pos
Opioid use, MME (mg)										
First in PPP	24	100.3 ± 123.2	7	77.1 ± 64.0	17	109.9 ± 141.2	0.82			
Last in PPP	24	36.6 ± 49.3	7	30.4 ± 28.7	17	39.1 ± 56.2	0.31			
At interview	25	31.1 ± 53.0	6	46.3 ± 63.4	19	26.3 ± 50.3	0.37			
Change in PPP	24	−63.8 ± 109.7	7	−46.8 ± 61.0	17	−70.8 ± 125.5	0.61	0.004 *	0.09	0.02 *
Change from first PPP visit to interview	23	−67.0 ± 148.2	6	−28.8 ± 103.7	17	−80.4 ± 161.6	0.94	0.03 *	0.29	0.05
Change from last in PPP to interview	23	−1.5 ± 70.6	6	21.7 ± 46.5	17	−9.7 ± 76.9	0.35	0.96	0.40	0.66

N: number; SD: standard deviation, * *p* < 0.05

**Table 10:** Self-reported pain-related outcomes at first and last PPP visits, with a two-group comparison between participants with positive and negative PPP experiences.

	All Study Participants			Negative Experience			Positive Experience			*p*-Values	
	N	Mean ± SD		N	Mean ± SD		N	Mean ± SD		Two-Group	Pre-Post
Pain severity											
First	24	5.54 ± 2.07		7	6.04 ± 1.36		17	5.34 ± 2.30		0.36	
Last	24	4.65 ± 2.58		7	6.61 ± 2.48		17	3.84 ± 2.21		0.02 *	
Change	24	−0.90 ± 2.19		7	0.57 ± 1.37		17	−1.50 ± 2.21		0.04 *	0.10
Pain interference											
First	24	6.70 ± 2.28		7	7.49 ± 1.69		17	6.38 ± 2.46		0.25	
Last	24	4.30 ± 3.19		7	6.80 ± 3.42		17	3.28 ± 2.53		0.02 *	
Change	24	−2.40 ± 2.46		7	−0.69 ± 2.20		17	−3.10 ± 2.26		0.03 *	<0.001 **
Pain Catastrophizing Scale											
First	24	21.54 ± 13.21		7	27.71 ± 13.47		17	19.00 ± 12.62		0.09	
Last	24	17.96 ± 15.30		7	31.00 ± 16.78		17	12.59 ± 11.21		0.02 *	
Change	24	−3.58 ± 11.16		7	3.29 ± 11.12		17	−6.41 ± 10.17		0.10	0.10
Insomnia Severity Index											
First	24	14.33 ± 9.07		7	13.14 ± 10.96		17	14.82 ± 8.50		0.68	
Last	24	12.79 ± 7.55		7	16.71 ± 7.89		17	11.18 ± 7.01		0.13	
Change	24	−1.54 ± 9.25		7	3.57 ± 12.57		17	−3.65 ± 6.91		0.31	0.17
SF-12: Physical Composite Scale											
First	25	33.62 ± 10.38		6	35.29 ± 12.20		19	33.09 ± 10.05		0.66	
Last	20	35.75 ± 10.34		5	36.17 ± 11.37		15	35.61 ± 10.40		0.93	
Change	20	3.67 ± 10.91		5	3.01 ± 4.50		15	3.89 ± 12.47		0.90	0.19
SF-12: Mental Composite Scale											
First	25	41.75 ± 10.50		6	33.96 ± 9.21		19	44.21 ± 9.85		0.04 *	
Last	20	44.88 ± 14.08		5	32.90 ± 9.59		15	48.88 ± 13.21		0.02 *	
Change	20	3.04 ± 11.02		5	−3.73 ± 15.09		15	5.30 ± 8.83		0.18	0.14
SF-36: Physical Composite Scale											
First	8	32.50 ± 10.31		1	34.11		7	32.27 ± 11.11			
Last	6	33.64 ± 11.45		0			6	33.64 ± 11.45			
Change	6	2.02 ± 4.05		0			6	2.02 ± 4.05			0.25
SF-36: Mental Composite Scale											
First	8	49.17 ± 14.32		1	60.21		7	47.59 ± 14.70			
Last	6	54.44 ± 8.57		0			6	54.44 ± 8.57			
Change	6	1.94 ± 7.30		0			6	1.94 ± 7.30			0.75

N: number; SD: standard deviation, * *p* < 0.05, ** *p* < 0.0001.

## Data Availability

The datasets used and/or analyzed during the current study are available from T.S. on reasonable request.

## Supplementary Materials

The following supporting information can be downloaded at: https://www.mdpi.com/article/10.3390/jpm14010031/s1, Supplementary S1: Interview Guide for PPP Participants.
