# Supplementary material for: Patient Experiences and Clinical Outcomes in a Multidisciplinary Perioperative Transitional Pain Service
Source: J Pers Med. 2023 Dec 26;14(1):31. doi: 10.3390/jpm14010031 (PMC10821325; doi:10.3390/jpm14010031)
Supplement: Supplementary file 1 [file jpm-14-00031-s001.zip › Supplement Interview Script.pdf]

Interview Guide for PPP Participants

Thank you for taking time to speak with us today. We are interested in learning about your experience in the Perioperative Pain Program and your suggestions on how we can further improve it. The questions we are going to ask you don't have right or wrong answers.

Your participation is strictly voluntary, and you are in no way required to provide information if you choose not to at any point. We are audio recording and taking notes during the interview so we can review what you have said at a later time. Our entire conversation today will be kept strictly confidential and no identifying information (i.e. your name or the patient's name we discuss) will be associated with your responses. The interview will take approximately 45-60 minutes. Do you have any questions before we begin?

Main Objectives:

Could you tell me about your general experience in the pain program?

- How many visits did you have with the pain program clinic?
- What did you find most useful about the pain program?
- Were there any matters that you wish the program had addressed differently?
- What do you feel might help to make things easier for you or someone else taking part in the program? [Probe to see if there is anything the project could change to help.]
- How did participation in the pain program affect your quality of life?
    - What were the program's effects on your interactions with your family, friends, and/or work?

What happened since your last pain program clinic visit?

- Did you resume opioids, and if so, are you currently on a higher dosage than when you were in the PPP?
- Did you transition to another pain clinic? Who is managing your pain now? (specialty, clinic, etc.)
- What were some of the challenges in pain management that you experienced after you left the pain program?
    - How did you handle those challenges?
- What pain management coping mechanisms are you still using?
- Do you have a mental health provider?
    - Is it the same provider you were seeing prior to your surgery, or is it Dr. Speed?

Were there any gaps in care between your treatment within the PPP and your current treatment (if any)?

- How did you manage your pain during that gap?
- How do you think the program might be changed to improve the transition from the pain program to another pain provider?
    - Do you think there is anything that your PPP provider could've done differently to ease the transition from their care to your current or another pain provider?

What were your main concerns about discharge from the pain program clinic?

- How did the pain program clinic help you address these concerns?
- What do you think we should know about the discharge process from leaving the pain program?
- Would you return to the pain program if you had another surgery? Why?

Closing remarks: is there anything else you would like to tell us about your experience?

If person indicated that they did not participate in the entire program (and this has not come up in previous questions):

- Tell us about the time when you chose to not return to a pain program clinic visit?
- What made you choose to do so?
- Was there anything that would have made you make a different decision?

Wrap-Up

That is the end of our interview today. Thank you so much for your time. Do you have any questions for me? [Answer any questions.] If you have any other thoughts or questions, feel free to contact me.
